# The FUS about SFPQ in FTLD spectrum disorders

**DOI:** 10.1093/brain/awaa207

**Published:** 2020-08-25

**Authors:** Rickie Patani

**Affiliations:** a1 Department of Neuromuscular Diseases, Queen Square Institute of Neurology, University College London, London, UK; a2 The Francis Crick Institute, London NW1 1AT, UK

## Abstract

This scientific commentary refers to ‘Aberrant interaction between FUS and SFPQ in neurons of a wide range of FTLD spectrum diseases’, by Ishigaki *etal*. (doi:10.1093/brain/awaa196).

**This scientific commentary refers to ‘Aberrant interaction between FUS and SFPQ in neurons of a wide range of FTLD spectrum diseases’, by Ishigaki *etal*. (doi:10.1093/brain/awaa196).**

Frontotemporal lobar degeneration (FTLD), amyotrophic lateral sclerosis (ALS), corticobasal degeneration (CBD) and progressive supranuclear palsy (PSP) have distinct clinical features from presenting symptoms through to management. However, accumulating evidence suggests some convergence in molecular and genetic pathology, which may argue against their taxonomy as completely distinct entities. Indeed these disorders are often referred to as the FTLD spectrum, for which fused in sarcoma (FUS), transactive response (TAR) DNA-binding protein 43 (TDP-43), and tau are pathological hallmarks (Seelaar *etal.*, 2011). TDP-43, FUS and splicing factor, proline- and glutamine-rich (SFPQ) are also recognized hallmarks of ALS-FTLD spectrum disorders. However, the precise nature of how these proteins might conspire to cause neurodegeneration has remained elusive. The aforementioned proteins, excepting tau, are canonical RNA binding proteins (RBPs), which are predominantly nuclear and regulate diverse aspects of RNA metabolism, including alternative splicing of pre-mRNA. Additionally, these RBPs have been found to be displaced from the nucleus in FTLD spectrum disorders (Neumann *etal.*, 2006; Luisier *etal.*, 2018; Tyzack *etal.*, 2019) raising the issue of aberrant pre-mRNA splicing through a loss of nuclear function as a possible underlying disease mechanism. Some of these RBPs have also been shown to accumulate and aggregate within the cytoplasm, which also then raises a gain of toxic function hypothesis. These two broad mechanisms are of course not mutually exclusive and indeed evidence exists for both across multiple model systems. However, the loss of an RBP from the nucleus is not a prerequisite for loss of its nuclear function. In their previous work, Ishigaki and co-workers used a mouse model to demonstrate that FUS and SFPQ normally co-localize to form high molecular weight complexes in the nucleus that regulate alternative splicing of microtubule-associated protein tau (*MAPT*) pre-mRNA, which encodes the protein tau. Disease-associated mutations in the *FUS* gene were sufficient to disrupt formation of the FUS-SFPQ complex, resulting in deregulated alternative splicing of *MAPT* pre-mRNA and a consequent increase in the 4-repeat (4R)-tau/3-repeat (3R)-tau (4R-T/3R-T) ratio, ultimately leading to neurodegeneration that recapitulated key aspects of FTLD (Ishigaki *etal.*, 2017). In this issue of *Brain*, Ishigaki and colleagues reason that similar deregulation of FUS-SFPQ complexes in neuronal nuclei may represent a more generalizable pathomechanism across human FTLD spectrum disorders (Ishigaki *etal.*, 2020).

Against this background, they systematically examined the intranuclear interaction between FUS and SFPQ in human tissue from 107 cases with FUS-, TDP-43-, or tau-related neurodegenerative disorders in addition to 35 control cases. They show co-localization by immunohistochemistry of FUS and SFPQ in the hippocampal granule cell nuclei of Alzheimer’s disease cases and controls. However, FUS and SFPQ were spatially dissociated in the FTLD spectrum disorders: ALS/FTLD-FUS, ALS/FTLD-TDP, PSP and CBD. They went on to confirm these observations in Betz cells of the primary motor cortex and orthogonally validated their findings using reciprocal co-immunoprecipitation assays. They additionally identified an increase in the 4R-T/3R-T ratio in ALS/FTLD-TDP and PSP cases compared to both Alzheimer’s disease and controls by quantitative PCR (Fig. 1) (Ishigaki *etal.*, 2020).

**Figure 1:**
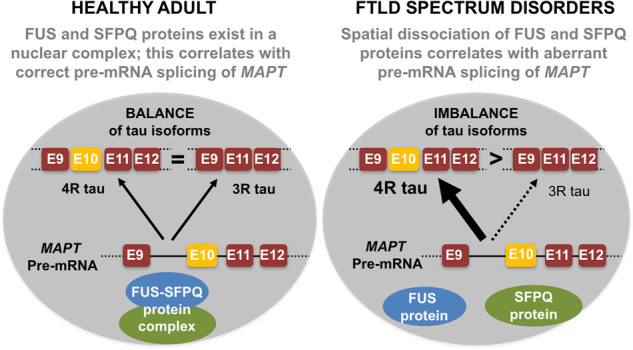
**Schematic depiction of proposed model for pathogenesis in human FTLD spectrum disorders.** In control tissue, the splicing factors FUS and SFPQ exist in a nuclear complex; this correlates with correct pre-mRNA splicing of MAPT, leading to a balance of 3R:4R isoforms. In FTLD spectrum disorders, there is spatial dissociation of FUS and SFPQ proteins, which correlates with aberrant pre-mRNA splicing of MAPT leading to 4R dominance and has been shown to be pathogenic in mouse models. 3R tau = 3 repeat tau; 4R tau = 4 repeat tau; E = exon.

The spatial dissociation of FUS and SFPQ in FTLD spectrum disorders may be exacerbated by nuclear loss of these two RBPs (Luisier *etal.*, 2018; Tyzack *etal.*, 2019). However, the paper published by Ishigaki and co-workers in this issue of *Brain* also reinforces the importance of considering intra-nuclear spatial dissociation of functional complexes in the molecular pathogenesis of FTLD spectrum disorders. These findings also raise the hypothesis that interactions between FUS and SFPQ are necessary for their stable nuclear localization. However, the relationship between expression levels of these two proteins and their ability to form functional complexes within the nucleus remains to be determined. Likewise, the mechanistic link between splicing alterations in *MAPT* mRNA yielding 4R-tau-dominance and neurodegeneration itself remains to be demonstrated in this (clinical) context. Notably, in their previous study, Ishigaki and co-workers demonstrated that both FUS-silenced and SFPQ-silenced mice similarly exhibited increased 4R-T/3R-T ratios along with FTLD-like behaviour, hippocampal neuronal loss and phosphorylated tau accumulation (Ishigaki *etal.*, 2017). Normalization of the 4R-T/3R-T ratio by 4R-T co-suppression rescued these phenotypes in the mouse model, implicating an altered 4R-T/3R-T ratio as a key mechanistic component of the phenotypes observed (Ishigaki *etal.*, 2017). Consistent with these data, an elevation of 4R-T in wild-type mice has been reported to cause abnormal behaviour (Schoch *etal.*, 2016). The temporal correlation (and indeed causation) between aberrant 4R-T/3R-T ratio and the aforementioned phenotypes will be important to resolve in future studies, specifically with respect to neuronal dysfunction versus actual neuronal loss. The precise mechanisms underlying how the reported splicing change in *MAPT* leads to aberrant total tau protein expression and the formation of characteristic inclusions also require further evaluation.

It is noteworthy that Alzheimer’s disease, which featured as a disease control in this study, did not exhibit an aberrant 4R-T/3R-T ratio and had similar FUS/SFPQ spatial localization to control cases. This of course supports the recognized distinct pathomechanisms and reinforces the specificity of the molecular hallmarks of FTLD spectrum disorders described by Ishigaki and co-workers (Fig. 1). TDP-43, FUS and SFPQ are components of paraspeckles, which are non-membrane delimited entities formed on the long non-coding RNA NEAT1_2 within the nucleus. Paraspeckles tend to be absent in healthy motor neurons but are thought to play a role in gene expression regulation when they are present. However, enhanced paraspeckle formation has been reported in FTLD spectrum disorders including both sporadic and familial ALS cases (Shelkovnikova *etal.*, 2018). It is therefore plausible that FUS and/or SFPQ could be (maladaptively) recruited into these ALS-related paraspeckle structures, which may disrupt their ability to form functional complexes within the nucleus. However, further investigation into the potential relevance of paraspeckle structures in FTLD spectrum disorders is required in future studies.

##  

### An integrated perspective

FTLD spectrum disorders have traditionally been considered as protein misfolding diseases characterized by the formation of (cytoplasmic > nuclear) protein aggregates. However, deregulation of RBPs such as TDP-43, SFPQ and FUS, and other aspects of RNA metabolism, are increasingly recognized as crucial players in these disorders. The mechanisms by which these defects in protein homeostasis and RNA metabolism conspire to cause neuronal degeneration are of key importance to resolve. Indeed, a common site of molecular convergence here is the ribonucleoprotein (RNP) complex, which is composed of both RNA and proteins (RBPs). RBPs implicated in FTLD spectrum disorders commonly contain prion-like low complexity domains (LCDs) and intrinsically disordered regions, which render them aggregation-prone. These RBPs possess the important physiological capacity to undergo liquid-liquid phase separation (LLPS), which refers to the condensation of molecules into liquid-like non-membrane delimited organelles (the aformentioned nuclear paraspeckles are an example of this phenomenon). LLPS likely occurs through tightly regulated prion-like polymerization driven by weak, transient molecular interactions between LCDs and other multivalent protein or RNA interaction domains. LLPS can be regulated by specific protein:protein, protein:RNA, and/or RNA:RNA interactions and is a molecular principle governing transient intracellular compartmentalization. LCD mutations can promote the fibrillization of molecular assemblies from (initially reversible) liquid-like droplets. Such perturbation in the kinetics of LLPS (i.e. hyperassembly and/or defective disassembly) may ultimately lead to some of the most recognized features of FTLD spectrum disorders including insoluble protein aggregates within the cytoplasm. Additionally, deregulated LLPS may lead to the sequestration of RBPs (including FUS, SFPQ and TDP-43), in turn causing their functional deficiency and the misregulation of key splicing events, such as *MAPT* pre-mRNA processing. Importantly, properly regulated LLPS is also crucial for the ability to concentrate specific proteins and/or RNAs at particular sites within the cell at the times when this is required for cellular homeostasis. This is a highly active research area and future studies addressing the spatiotemporal regulation of LLPS in different forms of neurodegneration are crucial in realizing the prospect of therapeutically targeting this fundamental process for patient benefit.

Specifically, future studies might aim to extend the important findings by Ishigaki and co-workers by addressing the potential relationship between spatial dissociation (and/or mislocalization) of RBPs and deregulated LLPS. Another key consideration is the cell type specificity of such molecular events, particularly with respect to glia given their well recognized roles in neurodegeneration. Furthermore, it would be important to understand whether the molecular phenomena reported in this study relate in some way to the increasingly recognized prion-like spread of pathology between cells in these disorders (Taylor *etal.*, 2016; Smethurst *etal.*, 2020). Unravelling the molecular and cellular ‘phases’ of neurodegeneration using orthogonal experimental models will ultimately guide the development of mechanistically rationalized therapies targeting salient pathogenic processes in the correct cell type(s).
